# Social anxiety disorder in an adolescent with agenesis of the corpus callosum: a case report

**DOI:** 10.1186/s12888-022-04332-0

**Published:** 2022-11-16

**Authors:** Runnan Yang, Yuan Cao, Danmei He, Wen Dang, Changjian Qiu, Wei Zhang

**Affiliations:** 1 Mental Health Center, West China Hospital, Sichuan University, 28 Dianxin Street, Chengdu, Sichuan China; 2 Department of Nuclear Medicine, West China Hospital, Sichuan University, Chengdu, Sichuan China; 3 Huaxi MR Research Center (HMRRC), Department of Radiology, West China Hospital, Sichuan University, Chengdu, Sichuan China; 4 West China Biomedical Big Data Center, West China Hospital, Sichuan University, Chengdu, Sichuan China

**Keywords:** Agenesis of the corpus callosum, Social anxiety disorder, Social phobia, Psychiatric disorders, Case report

## Abstract

**Background:**

The agenesis of corpus callosum (ACC) could impair the connectivity of the hemispheres of the cerebral cortex and cause cognitive impairments, social and behavioral issues, and even psychiatric disorders. Although social deficits are common in ACC patients, it is rare for a social anxiety disorder to occur.

**Case presentation:**

To report a 17-year-old adolescent with complete ACC associated with social anxiety disorder, depression, impulsive behavior, and other neurodevelopmental defects such as intellectual disabilities. His avoidance and fear were improved after treatment with sertraline.

**Conclusions:**

This is the first report of social anxiety disorder in ACC patients. The possible relationship between brain structural abnormities and anxiety syndrome should be investigated in more studies.

## Background

The corpus callosum, consisting of over 190 million axons, is like a bridge connecting the two cerebral hemispheres, which is associated with transferring and integrating meaningful information of the brain [1]. The agenesis of the corpus callosum (ACC) is a rare congenital anomaly with the prevalence varied from 0.02% to 0.7% in the general population [2–5]. Depending on the extent of absence, it can be divided into total or partial agenesis of the corpus callosum. According to its imaging manifestation, the ACC can be divided into the complex ACC when additional abnormalities, such as absent cavum septum pellucidum (CSP), ventriculomegaly, gray matter heterotopia (GMH), cerebellar abnormalities, polymicrogyria [6–9] were detected, and isolated ACC that no additional abnormalities were found [4]. The underlying risk factors of ACC can be, for example, gene mutations, disturbance of metabolism, intrauterine infections, and exposure to toxic substances such as alcohol [10].

The partial or total absence of corpus callosum can result in deficits in social functioning including emotion recognition, language comprehension, theory of mind [11], executive function impairment, and behavioral problems [10–13]. Besides, patients would be put at risk of mental retardation and have a worse outcome than isolated ACC if they had a complex ACC problem [14]. Some studies suggested that the prevalence of callosal disorders among children with intellectual disabilities was at approximately 2–3% [15–17]. Also, it’s worth mentioning that ACC syndrome may overlap with the profile of autism spectrum disorder (ASD) for they both display social, attentional, and behavioral problems. And experts had investigated that among children and adolescents with ACC, 35–50% of them showed obvious autistic symptomatology [18].

Until now, studies have reported the comorbidity of ACC with schizophrenia [1, 19–21], depression disorder [22, 23], bipolar disorder [24], and even personality disorder [24]. Notably, social difficulties existed in ACC and also are characterized in social anxiety disorder (SAD). The key features of SAD are intense fear of social situation and anxious about being negatively evaluated [25]. It affects more subjective distress in male compared to female [26, 27]. The etiology of SAD can be associated with brain structural and functional changes (e.g., overactive of fear circuit) [28, 29]. Nonetheless, a link between SAD and ACC has not been reported yet. Thus, it is of great importance to report this case.

## Case presentation

A 17-year-old teenager was admitted to our hospital for unwillingness to interact with others and associated with a depressed mood. He was a full-term natural infant at his birth without a family history of psychiatric disorders. He was a left-behind child raised by his grandparents and sisters. Family members had noticed that the boy was an introvert and hardly interacted with his classmates when he was in elementary school. Besides, he shows poor academic performance which might be due to his learning difficulties. At the age of 14 years old, the behavior problem showed up. He took things away from stores without paying for them several times but had no awareness of the faults. The store owner once called the police, and then his father beat him hard. Since then, he began to be withdrawn and reluctant to go to a place where there were many people. He feared his inappropriate behavior would allow him to be judged and punished again. He often covered his eyes with his hands so that he can avoid eye contact when he met unfamiliar people. He usually stayed at home alone playing video games. He was diagnosed with autism spectrum disorder at the age of 15 years old and received sertraline and risperidone for treatment. However, he still feared interacting with unfamiliar people but was able to communicate with his mother or sisters. Also, he easily showed irritability and hostility towards his father. After graduating from junior high school, he moved to the city where his mother living, and had a new environment to make a living. However, he turned to feeling worthless and had suicide ideation 2 months prior to admission.

On hospital admission, his general and neurological examination were normal. Two experienced psychiatrists observed nervous looks, blushed, avoiding eye contact, a decrease of speech, hypobulia, and depression. He showed shyness and tension and hid his face when sitting around with his teenage roommates. But he could have a normal conversation and be relaxed when staying with his sister alone.

**Fig. 1:**
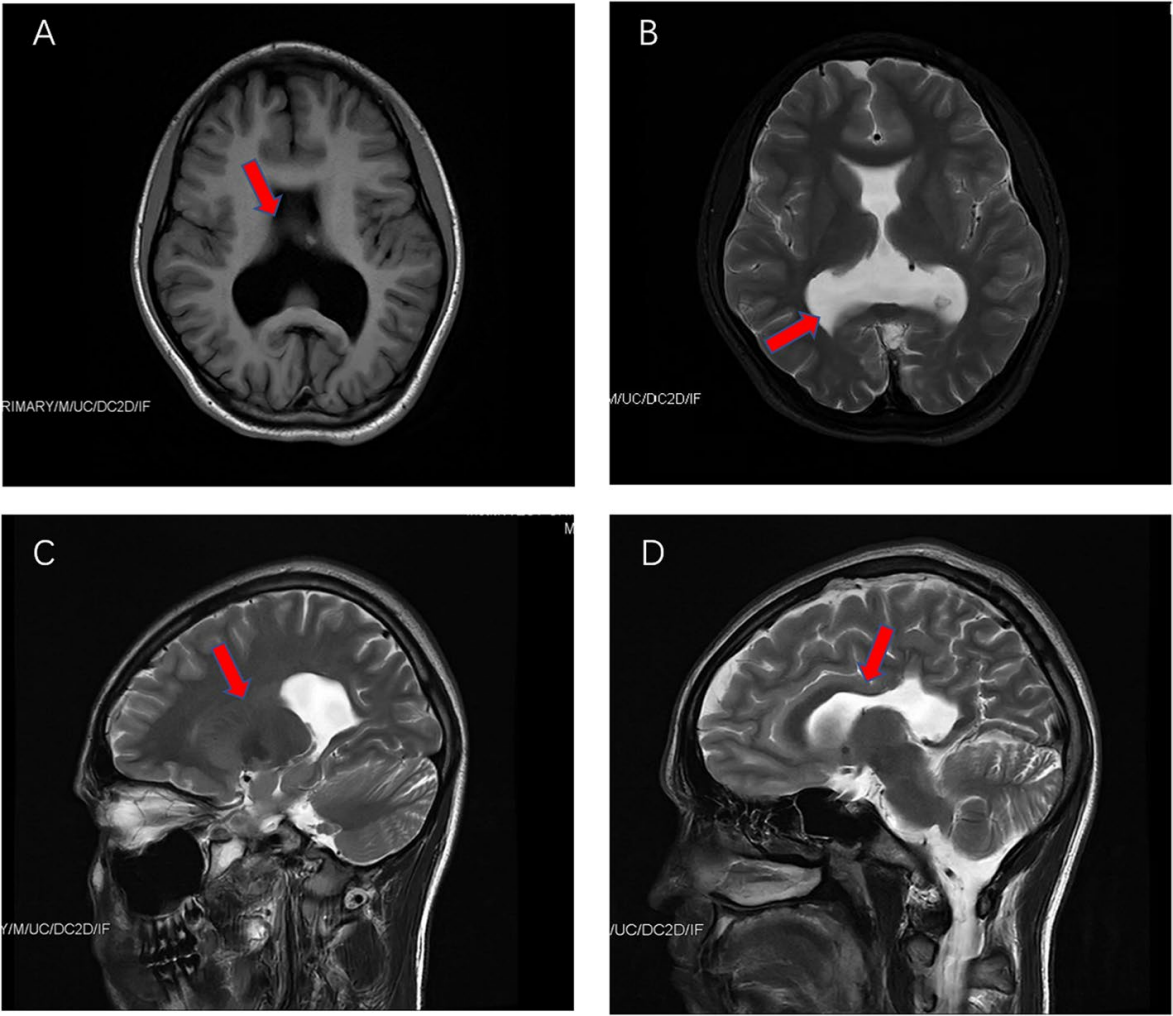
Brain MRI examination of a 17-year-old boy on admission. **A** and **B** axial T1-weighted images and T2-weighted images, showing absent septum pellucidum, connected lateral ventricles, and enlarged pedunculus posterior. **C** and **D** sagittal T2WI FLAIR images, presenting partial defect of the body of corpus callosum. And the cerebral convolutions adjacent to the absent corpus callosum region enlarged and shifted downward

Routine blood analyses were normal. The patient’s score on Raven's Standard Progressive Matrices was 21, which percentile rank was 4%, equal to Intelligence Quotient (IQ) score was 74. It suggested that the boy had intellectual disabilities. The Mini-Mental State Examination (MMSE) score was 18. The electrocardiogram (ECG) and electroencephalogram (EEG) were normal. Brain Magnetic resonance imaging (MRI) demonstrated a partial defect of the body of corpus callosum, absent septum pellucidum, and connected lateral ventricles. Besides, the short superior vermis of the cerebellum and upward displacement of the tentorium can be observed. Moreover, there were microgyria and polymicrogyria of bilateral frontal-parietal lobes. The shape of the sulci was also involved (Fig. 1).

During hospitalization, this boy showed marked anxiety and persistence avoidance that was provoked by social situations involving peers and adults. And fear of judgement by others for his inappropriate acting way. According to the fifth edition of the Diagnostic and Statistical Manual of Mental Disorders (DSM-5), he was considered as have a SAD [30]. Moreover, he has no restricted or repetitive behavior pattern and showed a reciprocal communication form when interacting with familiar people. Given this, we exclude the diagnosis of ASD. We began the administration of sertraline 50 mg per day to him. We observed the distinct social avoidance, he had a shy look and refused to interact when teenage patients invited him to play. Especially when we went rounds, he started covering his face with his hands and blushed, we could not have an effective conversation because he either rarely spoke or answered with “I don’t know, go ask my sister”. Still, he showed harmonious behavior and emotional reactions when playing with his sister. He was treated with sertraline 100 mg daily one week later. His mood brightened but the situation of social avoidance still existed.

One month after discharge, we learned via the follow-up phone call that the boy’s depressed mood lightened and his sister noticed that he no longer covered his face when walking on the street. He seemed more relaxed but still showed irritability to his parents. The boy had an outpatient visit two months after discharge. He rarely had eye contact with strangers, but he was not looking obvious sheepish and didn’t cover his face anymore. “I feel much better”, he answered us. We encourage him to conduct social skill-training with the assistance of his sister.

## Discussion and conclusions

Here we reported a 17-year-old Chinese juvenile who was suffering from ACC with comorbid social anxiety disorder, and accompanied by behavior problems, psychiatric symptoms. Individuals with ACC would suffer from social function deficiency. The ACC caused callosal connectivity reduction may result in the decrease of information processing and impair cognition development, thus limiting the capacity of the theory of mind [11], humor comprehension [31], facial expression understanding [32]. To explore anxiety disorders in ACC patients, we searched the ACC cases with anxiety published previously, and the summary is shown in Table 1. To the best of our knowledge, this is the first case of ACC with SAD.

**Table 1:** Review on previous reports of ACC patients with anxiety symptoms

Author (Year)	Age/Gender	Anxiety signs	Behavior problems	Other psychiatric disturbances	Intelligence level	Imaging performance	Associated with other Lesions
Russell [33] (1955)	19, Female	MMPI results: hypochondriasis; hysteria; psychasthenia	NA	depression; emotional instability; impaired concentration	Wechsler-bellevue test score: 61	complete absence of the corpus callosum	dilation of both lateral ventricles; Middle cerebral aqueduct the stenosis; the third ventricle was enlarged superiorly and posteriorly; the left cerebellar hemisphere was smaller than normal
Spak [34] (2019)	12, Male	generalized anxiety; aggression; stress	obsessive–compulsive disorder	attention deficit disorder; unable to concentrate on completing drumming and talking	IQ 68	partial agenesis of the corpus callosum(posterior)	NA
Párraga [35] (2003)	11, Male	irrational fears; anxiety attacks; separation anxiety; poor eye contact	hyperactivity; poor impulse control; difficulties relating to other children	visual and auditory hallucinations; marked distortions of reality; sleep difficulties	IQ 85	agenesis of the corpus callosum(unspecified)	colpocephaly
	10, Female	agitated movement	hyperactivity; temper outbursts; oppositional; lying; stealing; destruction	emotionality; sleep difficulties	IQ 102	partial agenesis of the corpus callosum (the rostrum and the body)	colpocephaly
David [24] (1993)	33, Male	fear; acute anxiety episodes	set fire; attack neighbors	mannerisms; hallucinations; delusions	IQ 78	partial agenesis of the anterior part of the corpus callosum	NA
Ernst [36] (1999)	15, Female	alexithymia; chronical pain; stressful events lead to an increase in pain	NA	NA	NA	agenesis of the corpus callosum(unspecified)	Chiari II Malformation; hydrocephalus

*Abbreviations*: *MMPI* Minnesota Multiphasic Personality Inventory, *NA* Not available, *IQ* Intelligence quotient

In terms of the pathological mechanism of social anxiety disorder, according to the research [25], poor social skills are independent risk factor of social anxiety. It especially may play a critical role in maintaining social phobia in children than it does in adults. Besides, genetic influence cannot be ignored. Prior twin studies and recent genome-wide association analysis (GWAS) study had revealed that SAD has a heritability basis [37, 38], shared genetic risk with extraversion. That is, children with high behavioral inhibition (BI), which refers to caution, fear, low rate of approach, and passive withdrawal in novel situations [39], may evolve to social phobia in their growth. ACC is often associated with those psychiatric disorders with social behavior deficits, such as autism and schizophrenia. The research of the complex mechanism of ACC suggested that the modifier genes that affect callosal formation may overlap with genes that lead to mental disorders [40]. And studies have shown that the risk of social anxiety in first-degree relatives of autism patients increased tenfold than that in relatives of patients with other neurodevelopmental disorders (e.g., Down's syndrome) [41]. It is possible that ACC disease significantly increased the risk of social anxiety disorder. Indeed, more research is needed to detect the possibility of comorbidity. What’s more, the changes of brain networks in both diseases seem to have something in common. The resting-state functional connectivity research of ACC patients demonstrated that impaired functional connection occurred in dorsolateral pre-frontal (DLPFC), posterior cingulate cortex (PCC), posterior parietal cortex (PPC), and parietal-occipital (PO) cortices regions [42, 43]. Alternatively, it is common that a reduced volume of the right ventral anterior cingulate gyrus and left inferior frontal gyrus in anxiety disorders [44].

There are some limits in this case. First, it was difficult to determine whether social deficits belong to primary ACC symptoms or the comorbidity of ACC. Second, we did not conduct a functional MRI test, which limits the richness of the results. Third, we are failing to follow patients for long-term so that we cannot acquire the condition changes and therapeutic effect. Finally, we collect history mostly depending on the verbal report of the patient’s family, which may not be able to explain his inner experience.

In summary, this is the first report of an ACC patient combined with social anxiety disorder. The relationship between structural abnormalities of the brain in stress-related disorder and congenital developmental disorder remains unclear. There is a need for prospective studies in the future.

## Data Availability

No datasets besides those reported in the article were generated during the current study.

## Abbreviations

ACC: Agenesis of corpus callosum
CSP: Cavum septum pellucidum
GMH: Gray matter heterotopia
ASD: Autism spectrum disorder
SAD: Social anxiety disorder
IQ: Intelligence Quotient
MMSE: Mini-Mental State Examination
ECG: Electrocardiogram
EEG: Electroencephalogram
MRI: Magnetic resonance imaging
DSM-5: The fifth edition of the Diagnostic and Statistical Manual of Mental Disorders
GWAS: Genome-wide association analysis
BI: Behavioral inhibition
DLPFC: Dorsolateral pre-frontal
PCC: Posterior cingulate cortex
PPC: Posterior parietal cortex
PO: Parietal-occipital
